# Residual flow may increase the risk of adverse events in patients received combined catheter ablation and transcatheter left atrial appendage closure for nonvalvular atrial fibrillation: a meta-analysis

**DOI:** 10.1186/s12872-019-1123-2

**Published:** 2019-06-10

**Authors:** Zhonglin Han, Xiang Wu, Zheng Chen, Wengqing Ji, Xuehua Liu, Yu Liu, Wencheng Di, Xiaohong Li, Hongsong Yu, Xinlin Zhang, Biao Xu, Rong Fang Lan, Wei Xu

**Affiliations:** Department of Cardiology, Nanjing University Medical School Affiliated Nanjing Drum Tower Hospital, Zhongshan Road No.321, Nanjing, 210000 China

**Keywords:** Left atrial appendage closure, Atrial fibrillation, Residual flow, Catheter ablation, Meta-analysis, One-stop therapy

## Abstract

**Background:**

Catheter ablation (CA) and left atrial appendage closure (LAAC) have been combined into a novel one-stop procedure for patients with atrial fibrillation (AF). However, postoperative complications are relatively common in patients undergoing LAAC; the complications, including residual flow, increase in the risk of bleeding, or other adverse events, are unknown in patients receiving one-stop therapy. Therefore, we tried to evaluate the adverse events of CA and LAAC hybrid therapy in patients with nonvalvular AF.

**Methods:**

We performed a meta-analysis and computer-based literature search to identify publications listed in the PubMed, Embase, and Cochrane library databases. Studies were included if patients received CA and LAAC hybrid therapy and reported adverse events.

**Results:**

Overall 13 studies involving 952 patients were eligible based on the inclusion criteria. In the periprocedural period, the pooled incidence of pericardial effusion was 3.15%. The rates of bleeding events and residual flow were 5.02 and 9.11%, respectively. During follow-up, the rates of all-cause mortality, embolism events, bleeding events, AF recurrence, and residual flow were 2.15, 5.24, 6.95, 32.89, and 15.35%, respectively. The maximum occurrence probability of residual flow events was 21.87%. Bleeding events were more common in patients with a higher procedural residual flow event rate (*P* = 0.03). A higher AF recurrence rate indicated higher rates of embolism events (*P* = 0.04) and residual flow (*P* = 0.03) during follow-up.

**Conclusions:**

Bleeding events were more common in patients with a higher procedural residual flow event rate. However, combined CA and LAAC therapy is reasonably safe and efficacious in patients with nonvalvular AF. Further studies on the safety and efficacy of CA or LAAC alone are necessary in future.

## Background

Globally, atrial fibrillation (AF) is the most commonly observed sustained cardiac arrhythmia, particularly in patients with structural heart diseases [1]. According to the European Society of Cardiology guidelines for the management of AF [2], catheter ablation (CA) is a class I treatment recommended for AF. However, because of the high rate of AF recurrence, most patients who receive CA should undergo warfarin or non-vitamin K antagonist oral anticoagulant (NOAC) therapy to reduce the risk of ischemic stroke or systemic embolism.

Although warfarin and NOACs reduce the risk of ischemic stroke and systemic embolism, they are associated with an increased risk of bleeding events [3]. Device-based left atrial appendage closure (LAAC) is a therapeutic modality for stroke prevention in patients with nonvalvular AF, particularly in those with a higher risk of bleeding determined using HAS-BLED scores. Recently, Wintgens et al. [4] reported that CA and LAAC could be combined into a one-stop therapy that can provide a straightforward strategy in maintaining rhythm and preventing stroke. Similar results have been reported by other studies with a small sample size and clinical studies performed at a single center [5, 6]. In 2015, the expert consensus statement of the European Heart Rhythm Association/European Association of Percutaneous Cardiovascular Intervention [7] suggested that combining the two left atrial interventions is a valuable and practical approach because of the common aspects of trans-septal puncture, anesthesia, and anticoagulation therapy.

However, patients are still recommended to undergo warfarin or NOACs after LAAC for at least 45 days because of the endothelialization of the left atrial appendage. During this time, it is unknown whether patients who are suitable for LAAC and have high HAS-BLED scores are associated with a high risk of bleeding or all-cause mortality. Moreover, postoperative complications, such as residual flow, are relatively common in patients who undergo LAAC, and postoperative complications increase the risk of bleeding and other adverse events. To the best of our knowledge, no meta-analysis has reported the postoperative complications in patients with a high risk of bleeding using one-stop therapy. Therefore, to evaluate the efficacy and safety of this new strategy, we performed a comprehensive meta-analysis of the contemporary literature.

## Methods

The meta-analysis was performed according to the preferred reporting items for systematic reviews and meta-analysis statement and registered in the PROSPERO database, an International prospective register of systematic reviews. (CRD42018106746, https://www.crd.york.ac.uk/PROSPERO/display_record.php?RecordID=106746).

### Literature search

We performed a computer-based literature search to identify publications listed in the PubMed, Embase, and Cochrane library databases until August 31, 2018. The terms used for the literature search were as follows: [left atrial appendage] AND (occlu* OR Closure) AND ablation.

### Study selection

Eligible studies were independently selected by two authors. Studies or patients included in this meta-analysis were as follows: (1) full text studies of randomized controlled trials, prospective (PC), or retrospective (RC) studies; (2) patients with nonvalvular AF in studies who received combined CA and LAAC therapy; and (3) the sample size in the enrolled studies was at least ten. Embolism events in the enrolled studies and associated cases included ischemic stroke or systemic embolism. Exclusion criteria for the analysis were as follows: animal studies, conference abstract, case reports, review articles, meta-analyses, editorials, posters, and studies that did not provide primary outcome (all-cause mortality, residual flow events, bleeding events, embolic events, or AF recurrence during follow-up), or enough data to analyze the efficacy and safety. Disagreements were resolved via discussions.

### Quality assessment

The quality assessment of the eligible studies was independently performed by two reviewers and assessed using the methodologic index for nonrandomized studies (MINORS). To avoid the results being influenced by data from poorly conducted studies, only studies with more than eight points (noncomparative studies) or 12 points (comparative studies) were included in our analysis.

### Data extraction

Data were independently extracted by two investigators and disparities were resolved via discussions. The following characteristics pertaining to the study were extracted: author details; year of publication; patient age and sex; number of different clinical types of AF; patients receiving oral anticoagulant therapy; CHADS_2_, CHA_2_DS_2_-VASc, and HAS-BLED scores; size and type of LAA; size and the type of devices; and events during periprocedural and follow-up periods, including all-cause mortality, residual flow events, bleeding events, embolic events, and AF recurrence. We combined systemic embolism, ischemic events, device thrombosis, and systemic thrombosis into a single category called “embolism events.”

### Statistical analysis

The meta package (under the R environment, version 3.4.0.) was used for all statistical analyses. Summary results were presented as the incidence rate of the events (ratio of the number of events to patient number) and 95% confidence interval (CI). The main adverse events of all-cause mortality, residual flow events, bleeding events, embolic events, or AF recurrence during follow-up were analyzed. For the subgroup analysis, the effects of the most common adverse event and AF recurrence on other adverse outcomes were further analyzed, and the subgroups were divided based on the mean incidence rate of adverse events. However, all studies did not provide the exact data for each sample or standard deviation of mean rate of the events; therefore, the pooled mean value is shown without standard deviation. A *P* value of < 0.05 of I^2^ statistics was considered to indicate heterogeneity. If heterogeneity existed, a random-effect model was used to assess the overall estimate or a fixed-effect model was chosen. Because of the existence of extreme values, we calculated the “exact” Clopper–Pearson CI for the observed proportion [8]. Publication bias was assessed using a funnel plot via Egger’s test of the intercept. A *P* value of < 0.05 was considered statistically significant.

## Results

### Study selection and quality assessment

**Fig. 1:**
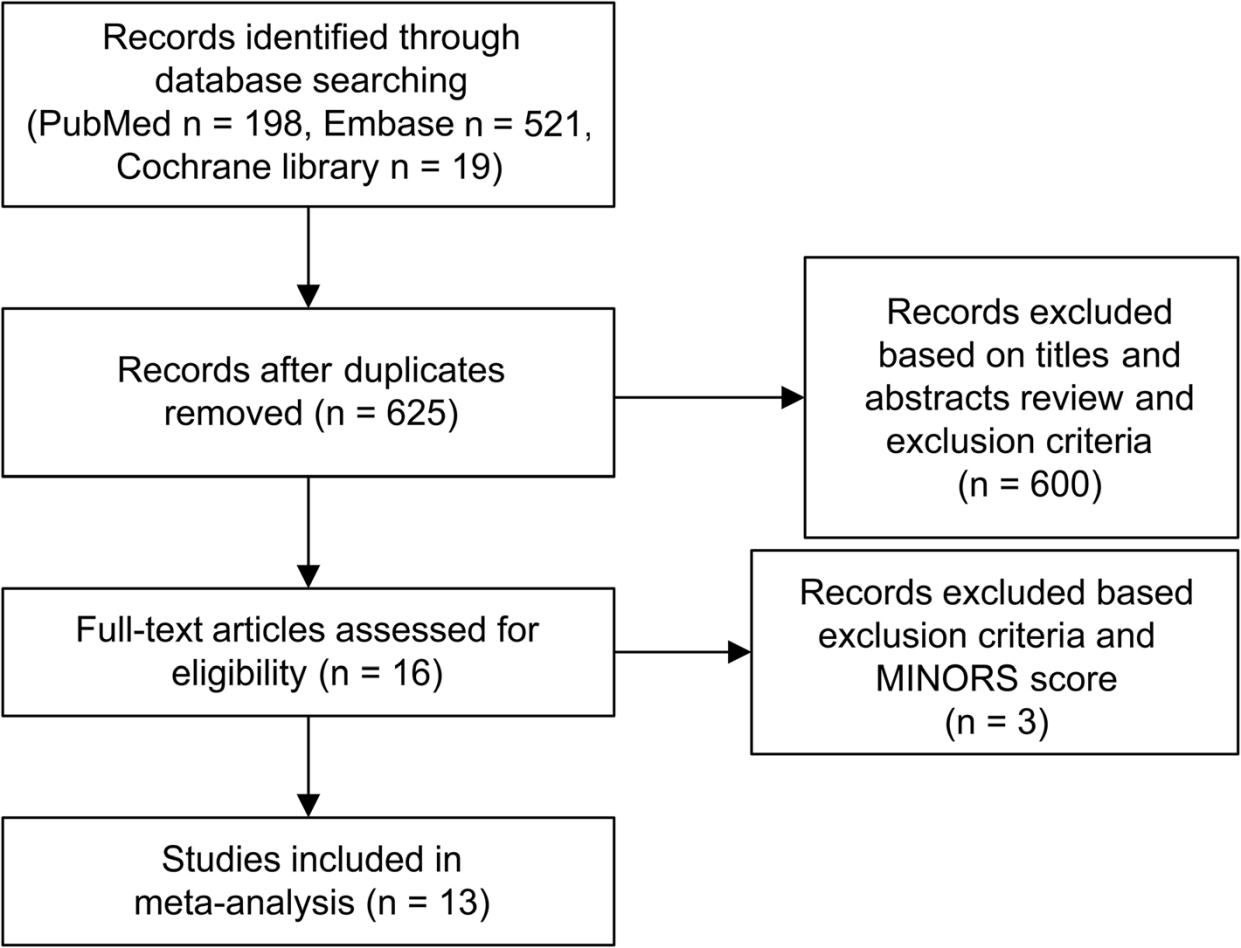
Flow chart of the study design

According to the search strategy, we searched 198 studies from PubMed, 521 from Embase, and 19 from the Cochrane library. After removing duplicates, 625 citations were identified. Moreover, after excluding nonrelevant studies, reviews, meta-analyses, conference abstracts, and case reports, 16 articles were identified (Fig. 1). Then, after quality assessment and full text review, 13 studies [4–6, 9–18] were finally enrolled in our analysis. Using the MINORS scores system, the scores of noncomparative and comparative studies were assessed between 8 and 12 and between 18 and 22, respectively (Table 1).

**Table 1:** Baseline characteristics and MINORS scores of the enrolled studies

Study	Patients (*n*)	Male (*n*)	Follow-up (month)	TEE follow-up (month)	Device	CHADS_2_	CHA_2_DS_2_-VASc	HAS-BLED	MINORS Score
Swaans 2012	30	21	12	6	Watchman	2.5	3	2	12
Walker 2012	26	20	12	6	Watchman	1.9	2.6	–	12
Swaans 2013	10	5	6	1.5	Watchman	3	3.5	1.5	11
Alipour 2015	62	40	38	2	Watchman	2.5	3.0	2	12
Calvo 2015	35	25	13	3	Watchman/Amplatzer	2.01	3.1	3.1	12
Romanov 2015	45	28	24	6	Watchman	–	2.2	3.5	18^a^
Phillips 2016	35	28	24	12	Amplatzer/Watchman	–	3	3	12
Fassini 2016	98	67	26.73	12	Watchman	1.5	2.6	1.9	10
Panikker 2016	20	13	12	9	Watchman	–	3.1	2.5	22^a^
Pelissero 2017	21	14	14.93	14.93	Watchman/Amplatzer	–	2.8	3.2	12
Wintgens 2018	349	202	34.5	3	Watchman	2.0	3.0	3.0	10
Phillips 2018	139	76	1	1	Watchman	2.2	3.4	1.5	8
Du 2018	82	48	11.2	6	Watchman	–	4.4	3.5	12
Total	952	587	–	–	–	–	–	–	–
Mean			22.83	5.18	–	–	–	–	–

^a^Comparative study

### Baseline study characteristics

The data of 952 patients (587 males, 61.03%) were analyzed (follow-up range, 1–38 months). The clinical types of AF were reported for 940 patients: 524 (55.62%) were paroxysmal, 342 (36.31%) persistent, and 74 (7.86%) long-term persistent AF. A total of 891 (93.59%) patients with AF received warfarin (415/648) or NOAC (233/648) therapy. Total 145 patients experienced bleeding events (total, 624) prior to therapy, and 161 (total, 527) refused oral anticoagulants or required device implantation. CHADS_2_, CHA_2_DS_2_-VASc, and HAS-BLED scores ranged 1.5–2.5, 2.2–4.4, and 1.5–3.5, respectively, in different studies.

During the periprocedural period, 99.37% patients showed a successful seal. Mean widths (20.82 mm) and lengths (28.50 mm) of the LAA were reported for 477 and 240 patients, respectively. There was a multilobular atrial appendage in 39.58% patients. Mean total procedure, mean LAAC, and mean fluoroscopy time were 156.24, 43.21, and 27.10 min (901, 689, and 897 patients), respectively. The closure systems used in the studies were the Watchman (937/952) and Amplatzer Cardiac Plug device (15/952). The mean size of the LAAC devices was 25.47 mm in 484 patients.

The estimated mean follow-up was 22.83 months, during which a 38.97% rate (not adjusted with a random effects model) of AF recurrence was documented among 793 patients, and repeated ablations were documented in 95 patients. The number of patients taking anticoagulant drugs decreased from 891 (93.59%) to 86 (11.61%) after hybrid therapy. The baseline characteristics and MINORS scores of the enrolled studies are shown in Table 1. The *P* value of all Egger’s linear regression tests for the baseline characteristics was > 0.05.

### Efficacy outcomes

**Fig. 2:**
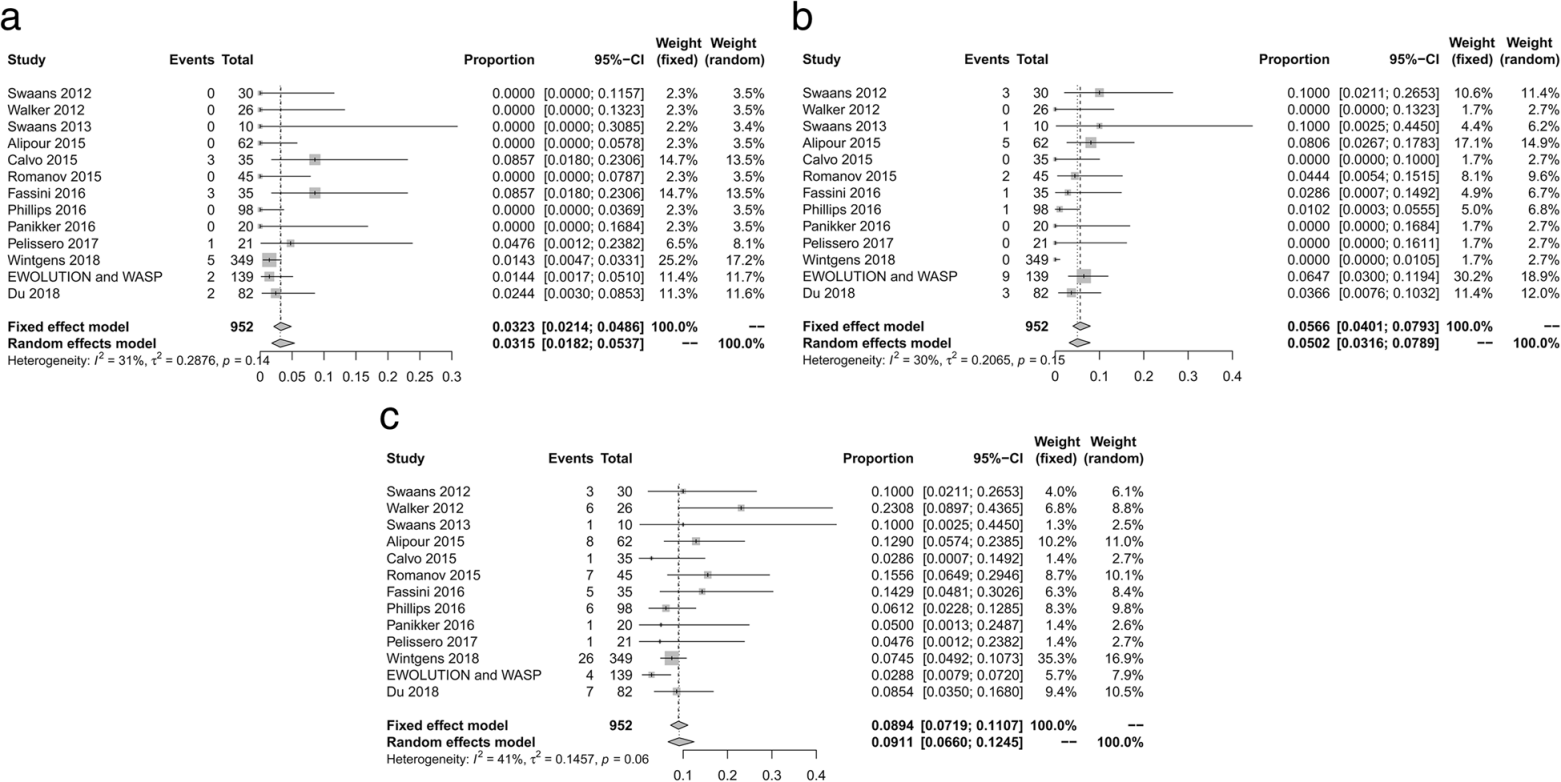
Pooled data of efficacy outcomes during the periprocedural period. (**a**) Pericardial effusion (**b**) Minor or major bleeding events (**c**) Residual flow event

In this meta-analysis, all outcome incidences are shown as an adjusted rate, which was calculated using a fixed or random effects model. In the periprocedural period, the pooled incidence of pericardial effusion was 3.15% (fixed-effect model, 95% CI, 1.82–3.57%). The incidences of minor or major bleeding events and residual flow observed were 5.02 and 9.11% (fixed-effect model, 95% CI, 3.16–7.89% and 6.06–12.45%), respectively. The pooled rates are shown in Fig. 2a–c.

**Fig. 3:**
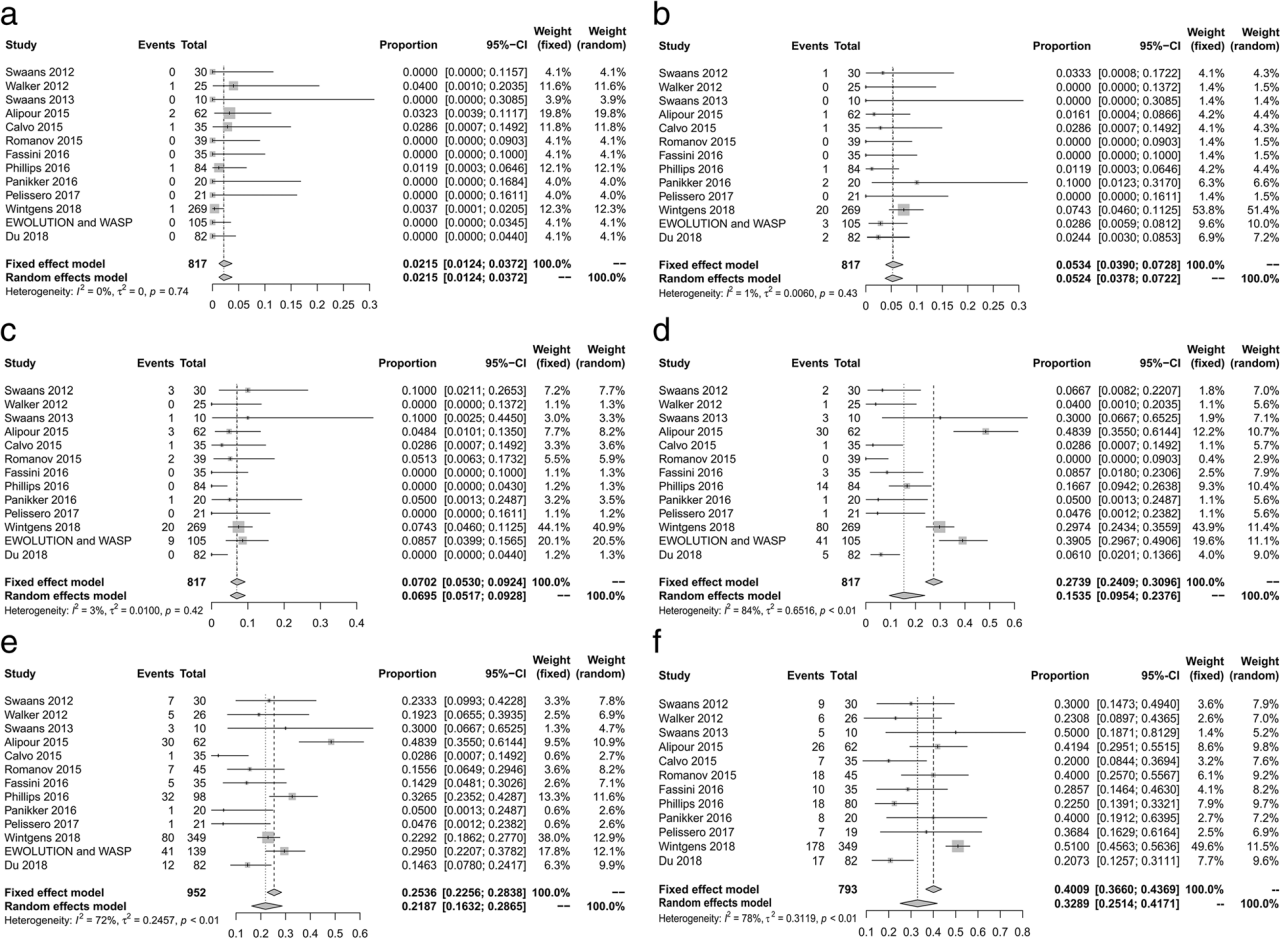
Pooled data of efficacy outcomes during follow-up. **a** All-cause mortality (**b**) Embolisms (**c**) Bleeding events (**d**) Residual flow events (**e**) Maximum occurrence probability of residual flow events (**f**) AF recurrence

During follow-up, the overall rate of all-cause mortality was 2.15% (fixed-effect model, 95% CI, 1.24–3.72%; Fig. 3a), but no one died of pericardial effusion. The incidence of embolism events (six patients received device embolization) and estimated incidence of bleeding events (seven major bleeding events) in the enrolled studies were 5.24 and 6.95% (fixed-effect model, 95% CI, 3.28–7.22% and 5.17–9.28%; Fig. 3b and c), respectively.

During transoesophageal echocardiography (TEE) follow-up (5.18 months), the incidence of residual flow events and maximum occurrence probability of residual flow events was 15.35 and 21.87% (random effects model, 95% CI, 9.54–23.76% and 16.32–28.65%; Fig. 3d and e) respectively. Furthermore, the average reported time of maximum events in the enrolled studies was 2.26 months, and the AF recurrence rate was 32.89% (fixed-effect model, 95% CI, 25.14–41.71%; Fig. 3f) during the follow-up.

### Subgroup analysis

According to the results of the meta-analysis, the most common adverse event was residual flow. In the subgroup analysis, we further analyzed the effects of residual flow during follow-up or TEE follow-up on other adverse outcomes. Procedural bleeding events were more common in patients with a higher residual flow rate (*P =* 0.03). Furthermore, residual flow may cause an increase in mortality; however, no significant statistical difference was observed (*P =* 0.12; Table 2). Maximum residual flow may cause more bleeding events; however, no significant statistical difference was observed (*P* = 0.08; Tables 3 and 4). Additionally, we found that a higher AF recurrence rate indicated a higher rate of embolism events (*P =* 0.04) and residual flow (*P =* 0.03) during follow-up, but it had no relationship with the maximum incidence of residual flow (*P =* 0.48; Table 5).

**Table 2:** Subgroups of procedural residual flow

Subgroup		Pool incidence (%)	95% CI (%)	*P*
Pericardial effusion	≤Mean	2.96	[1.47–5.85]	0.67
	>Mean	3.80	[1.49–9.36]	
Procedural bleeding event	≤Mean	2.75	[1.20–6.18]	0.03
	>Mean	7.85	[4.77–12.63]	
All-cause mortality	≤Mean	1.42	[0.66–3.04]	0.12
	>Mean	3.39	[1.53–7.35]	
Embolism	≤Mean	5.08	[3.11–8.18]	0.22
	>Mean	2.72	[1.14–6.38]	
Follow-up bleeding event	≤Mean	6.13	[3.70–9.99]	0.73
	>Mean	6.95	[5.17–9.28]	
Follow-up residual flow	≤Mean	14.80	[7.99–25.77]	0.87
	>Mean	13.39	[4.26–34.84]	
Maximum residual flow	≤Mean	20.24	[13.98–28.39]	0.58
	>Mean	24.26	[13.92–38.82]	
AF recurrence	≤Mean	30.94	[18.70–46.60]	0.55
	>Mean	35.90	[29.42–41.71]	

Mean, mean incidence rate of procedural residual flow; *AF* atrial fibrillation, *CI* confidence interval

**Table 3:** Subgroups of follow-up residual flow

Subgroup		Pool incidence (%)	95% CI (%)	*P*
All-cause mortality	≤Mean	2.65	[1.19–5.78]	0.47
	>Mean	1.70	[0.69–4.10]	
Embolism	≤Mean	4.19	[2.26–7.63]	0.93
	>Mean	4.35	[2.28–8.15]	
Follow-up bleeding event	≤Mean	5.46	[3.03–9.62]	0.36
	>Mean	7.51	[5.12–10.90]	
AF recurrence	≤Mean	28.88	[23.21–35.30]	0.17
	>Mean	39.98	[25.87–55.98]	

Mean, mean incidence rate of follow-up residual flow; *AF* atrial fibrillation, *CI* confidence interval

**Table 4:** Subgroups of maximum residual flow

Subgroup		Pool incidence (%)	95% CI (%)	*P*
All-cause mortality	≤Mean	2.77	[1.20–6.25]	0.42
	>Mean	1.76	[0.84–3.68]	
Embolism	≤Mean	4.08	[2.08–7.83]	0.71
	>Mean	4.78	[2.85–7.91]	
Follow-up bleeding event	≤Mean	3.93	[1.93–7.85]	0.08
	>Mean	7.87	[5.71–10.75]	
AF recurrence	≤Mean	28.82	[22.36–36.28]	0.22
	>Mean	38.10	[25.82–52.51]	

Mean, mean incidence rate of maximum residual flow during TEE follow-up period; *AF* atrial fibrillation, *CI* confidence interval, *TEE* transesophageal echocardiography

**Table 5:** Subgroups of AF recurrence

Subgroup		Pool incidence (%)	95% CI (%)	*P*
All-cause mortality	≤Mean	2.60	[1.17–5.68]	0.66
	>Mean	2.03	[0.91–4.45]	
Embolism	≤Mean	2.92	[1.47–5.74]	0.04
	>Mean	6.80	[4.53–10.09]	
Follow-up bleeding event	≤Mean	2.93	[0.98–8.44]	0.11
	>Mean	7.23	[5.10–10.16]	
Follow-up residual flow	≤Mean	8.33	[4.68–14.40]	0.03
	>Mean	21.65	[11.32–37.64]	
Maximum residual flow	≤Mean	18.85	[10.67–31.11]	0.48
	>Mean	24.45	[14.42–38.59]	

Mean, mean incidence rate of AF recurrence; *AF* atrial fibrillation, *CI* confidence interval

## Discussion

In patients with nonvalvular AF, the risk of stroke increases 5-fold compared with that in patients with sinus rhythm [19]. CA is an efficacious therapy for normalizing rhythm in patients with symptomatic AF. However, because of the high rate of AF recurrence, it is recommended that such patients additionally receive oral anticoagulant therapy. LAAC has emerged as an alternative to long-term anticoagulation therapy with warfarin or NOACs. LAAC has an efficacy similar to warfarin for all-cause stroke prevention [20]. Phillips et al. [17] pooled the data of patients with a high risk of stroke from real-world Watchman LAAC registries and demonstrated the feasibility and safety of combined CA and LAAC therapy. However, the long-term outcomes of this new therapeutic strategy have not been well established. Accordingly, single-center clinical studies [6] have been performed, but the sample size used in these studies is relatively small. Postoperative complications, such as residual flow, are relatively common and influence reportedly prognosis in patients who undergo LAAC. Thus, we performed this meta-analysis to study the postoperative complications and outcomes of the combined CA and LAAC therapy.

The combined therapy has been receiving increasing attention as a potentially important treatment strategy; therefore, we concentrated on the primary adverse events, such as pericardial effusion and bleeding events. Although there are no comparative study data on one-stop therapy unlike CA or LAAC alone, these results were interestingly similar to those recently reported [21] on the safety and efficacy of LAAC alone; however, the rate of bleeding events was lower than that reported in our meta-analysis. The reason for this discrepancy might be the routine use of anticoagulants during the CA periprocedural period. Minor bleeding events accounted for 84% of the total events in our pooled data, and no patient died of pericardial effusion during follow-up. These results support the conclusion that the combined CA and LAAC therapy is safe during the periprocedural period.

Thrombus is located at the LAA in approximately 90% patients with nonvalvular AF [22]. Moreover, LAAC [23] and surgical LAA excision [24] are effective ways to prevent stroke in patients with AF. However, Noelck et al. [25] reported that LAA devices are associated with high rates of procedure-related harm and thus require further evaluation. In our analysis, the most common adverse event during the periprocedural period was residual flow (rate, 9.11%). However, it still remains controversial whether residual leakage can cause adverse cardiac events. Although the findings of the PROTECT AF trial and other studies did not establish a relationship between the minimal residual flow and adverse events, including thromboembolism [26, 27], and are not associated with other adverse cardiac events [28], these results are in contrast with those of the surgical literature, in which residual flows are associated with thrombus formation and other adverse clinical events [29, 30]. In addition, the results of recent studies suggest that residual flow or leak may result in a predisposition to device-related thrombus [31, 32]. In the present analysis of the periprocedural subgroup, we found a potential relationship between a high incidence of bleeding events and residual flow (*P =* 0.03). Meanwhile, patients in the subgroup with a higher periprocedural residual flow rate may have a higher possibility of all-cause mortality than those in the subgroup with a lower periprocedural residual flow rate (3.39% vs. 1.42%, *P =* 0.12); however, no patient directly died of residual flow. Minimal residual flow (< 5 mm) does not require special treatment or clinical intervention [20, 33]; however, the proportion of patients with high residual flow (> 5 mm) was very low, and the data in these studies were based on a limited number of events and follow-up period. In the present study, we found a higher incidence of bleeding events in patients with high residual flow. One potential treatment option for this includes the continuation of a course of warfarin or NOACs therapy for at least 45 days; furthermore, patients may continue the anticoagulation therapy if major residual flow (> 5 mm) is detected. Additionally, minor or major residual flow in the perioperative period was not related to their number during the average TEE follow-up (5.18 months) and average maximum-event reported time (2.26 months). Interestingly, the endothelialization of LAA may take 45 days and reduce the reflux of the LAAC device [34]; however, the actual time may be longer than we previously estimated. Interestingly, a recent study enrolled patients who received one-stop therapy, and the results demonstrate that the combination strategy is independently associated with the new peri-device leak [18]. Further research is needed to evaluate the possibility that CA leads to the lengthening of the endothelialization process. In summary, residual flow in the perioperative period does not lead to a poor prognosis, but the potential perioperative bleeding risk during continuous anticoagulant use should be of concern. In addition, transesophageal echocardiogram assessment is recommended at 6-month intervals if any peri-device leak is documented [18]. Furthermore, the relationship between residual leak and thrombus or other cardiac adverse events should be studied for advancement in clinical research, especially for patients with major residual flow (> 5 mm) and those who have received one-stop therapy.

In the present study, the mean follow-up was approximately 2 years, and the rate of all-cause mortality was 2.15% during this time. The incidence of bleeding and embolism events was 6.95 and 5.24%, respectively (follow-up period of approximately 2 years). In our pooled data of 50 bleeding events, only seven patients experienced significant bleeding events, such as bronchiectasis, carcinoma, Rendu–Osler–Weber disease, gastrointestinal bleeding, knee haematoma, hematuria, and groin haematoma, and no patients experienced or died from haemorrhagic stroke. The reason for this might be the marked decrease in the use of anticoagulant drugs after hybrid therapy (from 93.59 to 11.61%). A total of 31 embolism events occurred during the follow-up period; only six patients directly received device embolization. Although it was unclear if there was a relationship between device-related thrombus events and device embolization based on our pooled data, a previous meta-analysis [35] demonstrated that the rates of all-cause stroke/systemic embolism and ischemic stroke/systemic embolism increased in patients who developed device-related thrombus events, and the rates of haemorrhagic stroke also increased in these patients. Thus, further research is required to determine the association between implant device and embolism occurrence.

One issue is the tendency of higher peri-procedural residual flow or embolism that could be related to a concomitant inflammatory process promoted by ablating the left pulmonary veins. However, the risk of these major adverse events was seemingly lower than the overall risk of stroke by approximately 5% per year in patients with nonvalvular AF. Our data with regard to adverse events were similar to those reported by the early stage of PROTECT AF study [20], which in turn were similar to those of the previous PREVAIL and PROTECT AF trials and of some recently published meta-analyses [36, 37]. After the perioperative period, we further analyzed the relationship between the presence of residual flow during TEE follow-up and other adverse events. The subgroup analysis suggested that increased residual flow rate leads to a higher rate of bleeding events (*P =* 0.08); thus, patients with residual flow during TEE follow-up may have higher risk of bleeding events. Importantly, the presence of residual flow on TEE follow-up was not statistically associated with other adverse events during follow-up, including all-cause mortality, bleeding or embolism events, and AF recurrence. Thus, the safety and efficacy of combined CA and LAAC treatment were acceptable in our study.

Recently, the Watchman and Amplatzer devices have demonstrated a high rate of device-related thrombosis as reported by Fauchier et al. [38], and this drew the attention of researchers. They found that the incidence of device-related thrombosis in patients with LAA imaging was 7.2% per year and that of ischemic stroke was 4.0% per year. These results were significantly higher than those reported in previous studies [39] as well as in the present study. These discrepancies could be caused by a number of factors. Oral anticoagulation at discharge was considered to be a protective factor, but the proportion of patients who received oral anticoagulation was much lower than that in our study (33.47% vs. 93.59%). Moreover, the CHA_2_DS_2_-VASc score in our study was 2.2–4.4, much lower than the mean value in the study by Fauchier et al. (4.5 ± 1.5) [38].

AF recurrence after CA is a significant problem because the percentage of patients who are free from AF recurrence is not satisfactory [40]. The most common method of ablation is pulmonary vein isolation; however, the rate of AF recurrence was as high as 32.89% during the follow-up in our meta-analysis. In the subgroup analysis, patients with a higher AF recurrence rate also had a higher risk of embolism (*P =* 0.03) and residual flow (*P =* 0.04). These results indicate the potential effect of long-term residual leak on hemodynamics that may influence the maintenance of sinus rhythm. Conversely, complete occlusion seems to reduce the incidence of AF recurrence [41]. Electrical isolation of the LAA may reduce AF recurrence [42]. It was believed that the effects of complete occlusion on electrical remodeling of the LAA would provide a new method for the comprehensive treatment of AF in future.

Finally, compared with NOACs, LAAC delivers the expected results, making it a better cost-effective treatment strategy for the secondary prevention of stroke in patients with AF since the last 10 years [43]. Meanwhile, serious bleeding risk associated with oral anticoagulant continuation after ablation success seems to outweigh the benefits of thromboembolic risk reduction [44]. Based on the high bleeding events, it seems that one-stop therapy has better cost-effectiveness ratio in patients with a higher rate of bleeding risk. However, more randomized studies on the cost-effectiveness of this one-stop therapy are warranted to test our results.

### Study limitations

Most studies enrolled in our analysis had a small sample size and were single-center trials. The length of follow-up in different studies was reported as a mean value or median data; thus, we pooled the data to obtain a mean value, which may affect the accuracy of this component of our analysis. The discrepancy in the follow-up duration in different clinical trials may be an important confounding factor of clinical outcomes. Furthermore, the adverse effects of the shape of the LAA and interaction between some anti-arrhythmia drugs and anticoagulants on embolism and bleeding events were unclear in our analysis. Finally, most of the studies were noncomparative in nature. There remains a lack of comparative study data on one-stop therapy and standard oral anticoagulant therapy after AF ablation. Therefore, further comparative studies need to be designed in future.

Although there remains a lack of comparative study data, most primary adverse events, except the rate of bleeding events, were similar to those reported on the safety and efficacy of LAAC alone. Furthermore, we found that bleeding events were more common in patients with a higher procedural residual flow event rate. Patients receiving hybrid therapy with higher AF recurrence rate may have a higher rate of embolism events and residual flow during follow-up. Finally, our analysis suggests that combined CA and LAAC therapy is reasonably safe and efficacious in patients with nonvalvular AF. However, further studies on the safety and efficacy are needed to compare data with CA or LAAC alone.

## Conclusions

Our results demonstrate that the use of CA and LAAC in a single procedure is safe and efficacious. However, there is some risk of bleeding in patients with LAA residual flow when warfarin or NOACs are continuously used. Additionally, our results suggest that patients with AF recurrence suffer from embolism events or LAA residual flow during follow-up. Finally, the average reported time of maximum residual flow events suggests that the process of LAA endothelialization takes longer than that was previously thought, and more studies should be performed to evaluate whether CA can lead to a prolongation of device endothelialization.

## Data Availability

All data generated or analyzed during this study are included in this published article. And the studies enrolled in the manuscript are available in the [Pubmed] repository, [https://www.ncbi.nlm.nih.gov/pubmed]; [Embase], [https://www.embase.com/]; and [Cochrane], [https://www.cochranelibrary.com/cca].

## Abbreviations

AF: Atrial fibrillation
CA: Catheter ablation
CI: Confidence interval
LAA: Left atrial appendage
LAAC: Left atrial appendage closure
TEE: Transoesophageal echocardiography
